# The Spanish Body Image State Scale: Factor Structure, Reliability and Validity in a Colombian Population

**DOI:** 10.3389/fpsyg.2019.02553

**Published:** 2019-11-22

**Authors:** Moisés Mebarak Chams, Laura Tinoco, Dania Mejia-Rodriguez, Martha L. Martinez-Banfi, Hanna Preuss, Florian Hammerle, Jorge I. Vélez, David R. Kolar

**Affiliations:** ^1^Universidad del Norte, Barranquilla, Colombia; ^2^Departamento de Ciencias Básicas, Sociales y Humanas, Universidad Simón Bolívar, Barranquilla, Colombia; ^3^Grupo de Neurociencias del Caribe, Unidad de Neurociencias Cognitivas, Universidad Simón Bolívar, Barranquilla, Colombia; ^4^Department of Pediatric and Adolescent Psychiatry and Psychotherapy, Universitätsmedizin der Johannes Gutenberg-Universität Mainz, Mainz, Germany

**Keywords:** body image, caribbean population, reliability, factor analysis, eating disorders

## Abstract

**Objective:**

Body image is a construct highly dependent on culture and ethnicity. Furthermore, recent studies reveal that body image is not only a trait, but also a momentary state subject to change in diverse situational contexts. However, cultural influences on momentary body image have not been sufficiently investigated. To assess the influence of Latin American culture on momentary body image and to enable its comparison to Western countries, the Spanish translation of an existing state body image scale such as the Body Image States Scale (BISS) is needed. In addition, the factor structure, reliability and general validity of the Spanish BISS (S-BISS) should be evaluated prior to its application in diverse situational contexts.

**Method:**

We conducted a cross-sectional study evaluating 1137 individuals between the ages of 18 and 28 years from Barranquilla, Colombia, South America. The original BISS, which assesses body satisfaction, was translated from English to Spanish. Factorial structure, scale score reliability and convergent/divergent validity were assessed.

**Results:**

Exploratory and confirmatory factor analyses revealed that a one-factor model with correlated items best described the factorial structure present in the BISS questionnaire. The coefficient of scale score reliability was α = 0.92 (McDonalds ω = 0.93), with similar results for men and women. Significant differences between males and females were found with lesser body satisfaction in females (*W* = 163260, *p* = 0.016). Lower S-BISS scores indicating less body satisfaction were associated with higher BMI (*r* = −0.287, *p* < 0.001) and obtained in participants who were currently on a diet (*t*_1135_ = −3.98, *p* < 0.001). The S-BISS was negatively correlated with a trait body image measurement assessing body dissatisfaction (Body Shape Questionnaire, *r* = −0.577, *p* < 0.001) and a psychopathology questionnaire (Brief Symptom Inventory 53, *r* = −0.331, *p* < 0.001).

**Conclusion:**

The S-BISS is a valid and reliable instrument to assess body image in the Colombian population, and exhibits similar psychometric properties to those of the original version. Future studies should examine whether the S-BISS captures change in state body image when applied in diverse situational contexts.

## Introduction

Body image is defined as a multifaceted construct that refers to the way individuals experience and conceptualize their own body (Evangelista et al., 2016). Body image comprises three main interdependent dimensions that consist of (*i*) cognitive-emotional, (*ii*) behavioral, and (*iii*) perceptual properties (Cash et al., 2002). Although most research investigates body image as a trait being stable across a broad range of situations, body image and, more specifically, the satisfaction – dissatisfaction with one’s body image are subject to change by situational context or over time (Tiggemann, 2001; Cash et al., 2002). Capturing the situational and temporal change of body image has been and still is of importance in many research domains. For example, clinical psychologists might be interested in whether body satisfaction improves after a mirror exposure in the treatment of eating disorders or body dysmorphic disorder (Griffen et al., 2018), sports scientists might investigate immediate effects of physical activity on body image (Carraro et al., 2010), or media researchers might examine whether exposure to thin-ideal television commercials affects body dissatisfaction in adolescents (Hargreaves and Tiggemann, 2003).

Assessing the effects of situational contexts (e.g., location, company, temperature) as well as of time on any psychological constructs require instruments which can reliably and validly measure momentary states of these constructs. Still, many studies assessing the influence of situational context on body image use *ad hoc* created scales [e.g., visual analog scales, (Hargreaves and Tiggemann, 2003)] or merely modified and often shortened versions of trait measurements (Fitzsimmons-Craft et al., 2015), leaving room for improvement toward a reliable and valid measurement of momentary body image states (Thompson, 2004).

For this purpose, Cash et al. (2002) developed the Body Image States Scale (BISS) to measure momentary body image satisfaction. It is assessed by six Likert-type items asking the participant how they feel *right now* regarding, for example, their physical appearance, body size and shape, or physical attractiveness. Each statement offers a variation of nine degrees from satisfaction to dissatisfaction or vice versa, and an average score of momentary body satisfaction is computed. Surprisingly, as the BISS is comprised of these items capturing various domains of current body image, no factor analyses are reported and a general factor of body satisfaction is only assumed. In the original study, the BISS was distributed to psychology undergraduate students with the instruction to imagine themselves in five different positive or negative situational contexts and indicate how they would feel in that specific situation: (1) a neutral condition without any imagination, (2) a day on the beach, (3) alone in their bedroom looking at fashion magazines, (4) a party with friends, and (5) while stepping on a scale showing a weight close to their preferred weight. The BISS showed acceptable to good scale score reliability in women across all situations, but failed to achieve the same for men in the neutral and beach context. The temporal stability of the BISS was lower than trait measurements, but still in an acceptable range. Furthermore, the BISS correlated positively in both genders with a trait measure of body satisfaction, and negatively with body mass index (BMI) and trait measures of negative appearance schemas, body surveillance and body shame. Most importantly, significant effects of context and of context sex interaction on the BISS were found (less favorable body image in negative contexts for both sexes, but even stronger for women). To this day, the BISS is applied successfully in numerous studies assessing momentary body image and/or situational/temporal changes of body image; for example, exposure to experimental designs (Bell et al., 2007; Carraro et al., 2010; Halliwell et al., 2011), virtual reality paradigms (Mountford et al., 2015; Preston and Ehrsson, 2018) and experience sampling methods (Fuller-Tyszkiewicz et al., 2015, 2018; Thøgersen-Ntoumani et al., 2017).

Besides the importance of situational context on body image, body image and body satisfaction are also influenced by culture and ethnicity. Several studies suggest that body dissatisfaction and body image disturbances are associated with and prominent in countries with a more Western lifestyle (Holmqvist and Frisén, 2010), and, for example, exposure to Western television and the associated beauty standards may promote the increase of body dissatisfaction in Non-Western samples (Becker et al., 2002). Few differences in body dissatisfaction and body image were found between different ethnicities of women living in the United States (Grabe and Hyde, 2006) and no uniform patterns of body image concerns were found in males from different ethnic and cultural backgrounds (Ricciardelli et al., 2007). In recent years, socio-cultural research on body image laudably shifted from investigating mainly diverse United States American samples toward studies in diverse samples around the world (Cash, 2017), and especially in Latin America an increase in body image research was noted (Laus et al., 2014). This is accompanied by an increase in studies assessing the psychometric properties of body image measurements in different cultures and languages. Indeed, several instruments have been successfully translated to Spanish or Portuguese to assess *inter alia* body dissatisfaction (Castrillón Moreno et al., 2007), body checking/avoidance (Kachani et al., 2011; Campana et al., 2013) and positive body image (Swami et al., 2011; Alcaraz-Ibáñez et al., 2017) in Latin America.

Several studies then showed that the prevalence and sex differences of body dissatisfaction in Latin America are mostly similar to Western populations: for example, Mellor et al. (2008) showed that girls presented a greater body dissatisfaction than boys in Chile, and McArthur et al. (2005) found that 68% of females and 57% of males were dissatisfied with their body in a study conducted in six major Latin American cities in Guatemala, Argentina, Peru, Panama, Cuba, and Chile. This is more or less in line with newer studies from Chile (Oda-montecinos et al., 2018) and Brazil (Triches and Giugliani, 2007; Leite et al., 2014). Regarding Colombia, studies found that higher socioeconomic status is linked to obesity and higher body dissatisfaction in students from public schools (Gilbert-Diamond et al., 2009) found, and Borda Pérez et al. (2016) concluded there was a higher distortion in male students.

However, similar to body image research in Western countries, most research conducted in Latin America and other Non-Western countries focused on cultural differences in trait body image and body dissatisfaction. Cultural differences might not only exist in trait levels of body image, but also in the way people from diverse cultural backgrounds are affected by and deal with different situational contexts in relation to their momentary body image. For example, situational contexts such as a day at the beach similar to the situational cue used in the seminal Cash et al. (2002) study or a traditional dance might be less averse regarding the body image to people from Latin American countries than to Western individuals, as both situational cues are linked to the culture of Latin America. In addition, experimental studies measuring changes in body image after exposure to stimuli increasing ethnic identity might further enlighten the relation between body image and culture, as ethnic identity may serve as a buffer to thin-ideal media (Schooler and Daniels, 2014). Thus, translation of existing measures and/or the development of new measurements of momentary body image is needed to investigate cultural effects on momentary body image in experimental or naturalistic settings, and to compare such findings from Latin American populations with other countries. Only one study developed a Brazilian body satisfaction situation scale (Hirata and Pilati, 2010), which is not available in other languages. Since 2002, the BISS scale was at least translated to Italian (Carraro et al., 2010), Dutch (Alleva et al., 2014), and German (Vocks et al., 2007) for use in experimental investigations of body image, but so far, neither a Portuguese nor a Spanish version of the BISS exists. Before a translation of any questionnaire should be used in studies and conclusions drawn from the data, it is crucial to investigate whether the questionnaire is comparable to the original version. Therefore, a psychometric study is needed to investigate if the translated questionnaire measures the same construct on the same dimensions with the same reliability as the original version to avoid bias and to show equivalence of the measurements (Van de Vijver and Tanzer, 2004). In the case of the BISS, a first step is to investigate the factor structure and scale score reliability in a broad sample without differing situational contexts. Furthermore, the correlation of the BISS with several non-situational variables and related trait measures should be investigated. For example, any body image measurement would most likely show correlations with BMI, sex and age, and in the case of the BISS a high correlation with a trait body dissatisfaction measurement is expected. In addition, as body (dis)satisfaction is related to several mental illness, the correlation of the BISS with a measurement of psychological distress should be evaluated. Once the psychometric properties of a translated BISS are established, a second study can investigate whether the translated BISS is also capable of measuring body image differences in diverse situational contexts.

Therefore, the aim of this study is to translate the BISS from English into Spanish. Furthermore, we aim to assess the factor structure, sex invariance, scale score reliability, convergent validity and correlations with related constructs in a single cross-sectional sample to provide a starting basis for comparable assessment of momentary body image in Latin American populations.

## Subjects and Methods

### Participants

From May to August 2017, we assessed 1549 individuals mostly from the Colombian Caribbean coast, which is comprised of eight states [Atlántico, Bolívar, Córdoba, Sucre, Cesar, La Guajira, Magdalena, Antioquia (Urabá)] and has an estimated population of approximately 10.9 million habitants (Betín, 2018). The Colombian Caribbean Coast is a region where many populations and ethnicities converge [e.g., aboriginal Amerindian, African, and a complex admixture of European (Spain), Syrian-Lebanese, Sephardic Jew, German, Italian and English communities]. Recruitment was mainly performed in several undergraduate courses of different programs at the Universidad del Norte and Universidad Simón Bolívar, in Barranquilla, Colombia. Students were encouraged to invite friends and relatives to participate in the study, and were compensated with course credits for participation.

Of the initial 1549 participants, 236 participants did not complete at least one questionnaire, 26 data entries were identified as duplicates, and five cases were deleted as the response pattern suggested fake entries. To reduce noise attributable to cultural standards in body image across countries, only individuals being born in Colombia were included. Furthermore, individuals with a Body Mass Index (BMI; kg/m^2^) <40 and age in the 18–28 years range were included by applying Ueda’s method for outlier analysis (Marmolejo-Ramos et al., 2015), resulting in the exclusion of 145 additional individuals. Hence, the final sample was comprised of 1137 individuals.

Of these 1137 individuals, 718 (63.15%) were females and 419 (36.85%) were males. Age ranged from 18 to 28 years (mean = 20.9, median = 20, SD = 2.13) and differed between males and females (20.71 ± 2.01 vs. 21.34 ± 2.27; *t*_792_,_68_ = 4.683, *p* < 0.001). BMI averaged 22.7 ± 3.9 in the overall sample and was statistically different between males and females (23.6 ± 3.8 vs. 22.3 ± 3.9, *t*_901_,_33_ = 5.61, *p* < *0.001*). Most participants were currently enrolled as university students (*n* = 903, 79.42%), with 368 (40.75%) of them being psychology students. Another 114 (10.03%) participants were employed and 48 (4.22%) participants were currently unemployed, 12 (1.06%) were school students and 60 (5.28%) pursued other activities. Regarding sexual orientation, 1036 (91.11%) participants were heterosexual, 46 (4.05%) gay/lesbian, 48 (4.22%) bisexual and 7 (<1%) had another sexual orientation.

### Instruments

#### Body Image State Scale (BISS)

The BISS (Cash et al., 2002) consists of six items written to measure the following domains of body image at the moment of response: (1) dissatisfaction–satisfaction with one’s overall physical appearance; (2) dissatisfaction–satisfaction with one’s body size and shape; (3) dissatisfaction–satisfaction with one’s weight; (4) feelings of physical attractiveness– unattractiveness; (5) current feelings about one’s looks relative to how one usually feels; and (6) evaluation of one’s appearance relative to how the average person looks. Responses to each item were based on 9-point, bipolar, Likert-type scales, semantic anchored at each point. In the Spanish version, all items were presented in a positive-to-negative direction. Answers ranged from 1-a great deal (worse) to 9-a great deal (better), i.e., “Extremely dissatisfied” (9 points), “Mostly dissatisfied” (8 points), “Moderately dissatisfied” (7 points), “Slightly dissatisfied” (6 points), “Neither dissatisfied nor satisfied” (5 points), “Slightly satisfied” (4 points), “Moderately satisfied” (3 points), “Mostly satisfied” (2 points), and “Extremely satisfied” (1 points) with their physical appearance (Supplementary Material).

The BISS scale was translated with permission of the first author of the original study from English to Colombian Spanish by a bilingual Colombian psychologist, supported by another psychologist fluent in Spanish and English. The translation was piloted in a small test group of 20 undergraduate students and their suggestions were considered while revising the translated version of the BISS. Finally, the Spanish BISS (S-BISS) version was back-translated to English and compared with the original BISS scale, and adjustments were made according to observed semantical differences. The S-BISS is presented in the Supplementary Material. In line with the original BISS (2), a global average S-BISS score of body satisfaction was calculated ranging from 1 to 9.

#### Body Shape Questionnaire (BSQ)

The BSQ is a questionnaire for the assessment of cognitive-affective BD (Cooper et al., 1987). The BSQ originally contained 34 questions, but several shorter versions with good reliability (Cronbach’s α > 0.87 for all versions) and validity exist nowadays in Spanish and English (Warren et al., 2008). Therefore, the Spanish 8-item version BSQ-8c (Evans and Dolan, 1993) was used in this study with a good scale score reliability in our sample [α = 0.91; 95% confidence interval (CI) = 0.90–0.92]. The BSQ is used as a trait body dissatisfaction measure to be compared to the S-BISS scores in this study. Items were recorded as “Never” (1 point), “Rarely” (2 points), “Sometimes” (3 points), “Often” (4 points), “Very Often” (5 points), and “Always” (6 points), being a higher score an indicative of more body dissatisfaction.

#### Brief Symptom Inventory-53 (BSI-53)

The BSI-53 (Derogatis and Melisaratos, 1983) is a measure of general psychopathology, derived from the longer Symptom Check-List SCL90 by Derogatis and Cleary (1977). The BSI-53 showed good scale score reliability, test-retest reliability and convergent validity with other psychopathology measurements. In this study, the Spanish version of the BSI-53 by Pereda et al. (2007) was used, which also showed good scale score consistency in our sample (α = 0.97, 95% CI = 0.96–0.98). Although body dissatisfaction is predominantly linked to disordered eating, there is evidence that body dissatisfaction is an independent factor to psychological distress especially in adolescents and young adults (Johnson and Wardle, 2005). Therefore, we decided to use the BSI-53 as a measure of general psychopathology to see whether the S-BISS correlates with psychological distress. In the BSI-53, responses were recorded on a Likert-type scale with items being “Never” (1 point), “Rarely” (2 points), “Sometimes” (3 points), “Often” (4 points), “Very Often” (5 point), and “Always” (6 points). Thus, a higher score implies more psychopathology.

### Procedure

As part of a larger international project on body image in Colombia and Germany, all participants completed an online questionnaire, comprised of seven instruments regarding body image and related constructs, in ∼25 min. The order of the questionnaires was fixed, with the BISS being the second body image instrument, followed by the BSQ, and the BSI-53 being the last instrument assessed. The first included questionnaire was a translated version of the body image avoidance questionnaire – BIAQ (Rosen et al., 1991). However, it was not used in this analysis due to poor psychometric properties of the translation.

Data were collected anonymously and managed using REDCap^1^ electronic data capture software (Harris et al., 2009). Data were hosted at the University Medical Center of Johannes Gutenberg University Mainz, Germany. All individuals participated voluntarily, and university students were compensated with course credits for participation. Ethics approval was obtained prior to data collection from the internal Ethics Review Board at the Universidad del Norte, Barranquilla, Colombia (No. 155/2017) and from the ethical review board of the local medical association in Mainz, Germany (No. 837.336.16/10655). In line with the requirements of the ethical review boards, informed consent was obtained online as participants were presented with a detailed description of the study aims and were obliged to accept before continuing with the study.

### Statistical Analysis

All statistical analyses were performed in R version 3.3.3 (R Core Team, 2017). Mean values, ranges and standard deviations (SDs) were estimated for continuous variables; when required, normality was assessed using the Shapiro–Wilk’s test. For categorical variables such as gender and sexual orientation, frequencies and proportions were calculated. Potential differences between groups in frequency distributions were determined using a χ^2^-based test of independence. The Pearson’s linear correlation coefficient was used to determine potential correlations between pairs of continuous variables. Exploratory factor analysis was performed with the fa function of the ‘psych’ (Revelle, 2017) package for R with the default settings using varimax rotation. As for confirmatory factor analysis, the adequacy of the sample size was assessed using the Kaiser-Meyer-Olkin (KMO) statistic and the Bartlett’s test, both implemented in the “psych” package for R. Confirmatory factor analysis was performed using the “cfa” function of the “lavaan” package (Rosseel, 2012) for R with the default settings. Model improvement in confirmatory factor analysis was assessed using the modification index (MI), also known as LaGrange Multiplier or Score Test, as implemented in the R function with the same name in the “lavaan” package (here, higher MI values indicate a better fit of the corresponding model). The quality of the fitted factorial models was assessed using a battery of performance measures that included the Tucker Lewis index (TLI), the comparative fit index (CFI), the Bayesian information criterion (BIC), and the root mean square error of approximation (RMSEA). Considering that all residual-based indexes are generally less affected by sample size and model complexity than other indexes, we assumed that RMSEA values below 0.05, and TLI and CFI values greater than 0.95 suggest a close model fit (Schulz et al., 2011). Scale score reliability was assessed using Cronbach’s α and McDonald’s ω coefficient following previous recommendations (Peters, 2014). In addition, ω is a more accurate approximation of a scale’s reliability as Cronbach’s α is unrelated to the scale’s internal consistency and a defective reliability estimate (Peters, 2014).

Convergent validity of the S-BISS was assessed with the BSQ-8c to determine its relation to trait BD, and with the BSI-53 to examine the relation of the S-BISS with general psychopathology. S-BISS values of participants currently on a diet were compared to participants who were not as a way of assessing construct validity based on a known-group comparison. Lastly, means and standard deviations of the scale were calculated for separate gender and BMI categories.

## Results

### Exploratory and Confirmatory Factor Analyses

**FIGURE 1:**
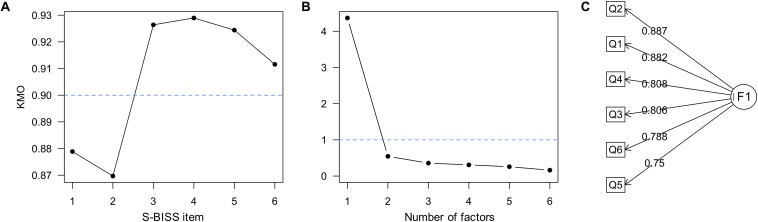
**(A)** Kaiser-Meyer-Olkin (KMO) test for individual items of the BISS scale. Values greater than 0.8 and 0.9 represent meritorious and marvelous sample adequacy to apply factor analysis on the data (Kaiser, 1974). **(B)** Scree plot for the BISS scale. Using the Kaiser criterion (blue line), a 1-factor structure is chosen. **(C)** Structure of the 1-factor model. Values next to labels Q1, Q2,…,Q6 correspond to the pattern matrix scores in Table 3. Abbreviations as in Figure 2.

The KMO test for sampling adequacy indicated that the sample was well suited to apply factor analysis (KMO ≥ 0.9), with good KMO indices in all items (average KMO = 0.91, SD = 0.026, range = 0.869 – 0.929; see Figure 1A). This result was subsequently confirmed using a Bartlett’s test applied to the correlation matrix ($\chi_{15}^{2}=5105$, *p* < 0.0001). Using the Kaiser criterion, that is all factors with eigenvalues greater than one (Costello and Osborne, 2005), we found that a 1-factor structure is sufficient (Figure 1B). Overall, this factorial model explained 73.4% of the total variance. Figure 1C depicts the structure of this model. As expected, this factorial structure is similar for males and females based on the TLI (0.918 in females vs. 0.927 in males), and the factorial loadings were positive. When testing whether scores are sex invariant, we found a significant increase in the χ^2^ statistic ($\chi_{12}^{2}=17.812$ vs. $\chi_{18}^{2}=47.7$; *p* < 0.0001), but not in the TLI, suggesting that the strong invariance assumption cannot be met.

Based on the results of our exploratory factor analysis, four different factorial models were proposed in our confirmatory factory analysis. The rational of these models is the improvement of the model fit based on the MI (see section “Subjects and Methods”). *Model 1* considers only one construct determined by all S-BISS items allowing no correlation between items; *Model 2* explores the same 1-factor structure of *Model 1* but allows correlation between items Q5 and Q6 (that is, Q5∼Q6); *Model 3* is similar to *Model 2* but allows the possibility that, in addition, items Q4 and Q6 are correlated (that is, Q4∼Q6 and Q5∼Q6); and *Model 4* is similar to *Model 3* and allows an additional correlation between items Q4 and Q5 of the S-BISS questionnaire (that is, Q4∼Q6, Q5∼Q6, and Q4∼Q5). The main results are presented in Table 2. According with the predefined performance measures, *Model 4* was selected as it performs exceptionally well based on the battery of performance measures previously described (Table 2). The selected model suggests that one factor with correlated items best describes the factorial structure of the S-BISS questionnaire. Table 3 shows the factor (pattern) matrix for *Model 4*, suggesting that the variance of each item is fairly explained by the 1-factor structure model.

### Scale Score Consistency and Reliability

Considering that the exploratory and confirmatory factor analyses showed that a 1-factor structure better explains the factor structure of the S-BISS scale, we proceeded to calculate the coefficient of scale score reliability and McDonald’s ω coefficient. For the complete S-BISS scale, the Cronbach’s α coefficient was 0.92 (95% CI = 0.91–0.93, *n* = 1137), with similar results for males (α = 0.92; 95% CI = 0.91–0.93, *n* = 419) and females (α = 0.93; 95% CI = 0.92–0.94, *n* = 718), while the ω coefficient was 0.926 (95% CI = 0.917–0.938) in the full sample, and 0.929 (95% CI = 0.914–0.935) and 0.918 (95% CI = 0.910–0.938) for females and males, respectively.

### Relationship Between S-BISS, Gender, Age, and BMI

**FIGURE 2:**
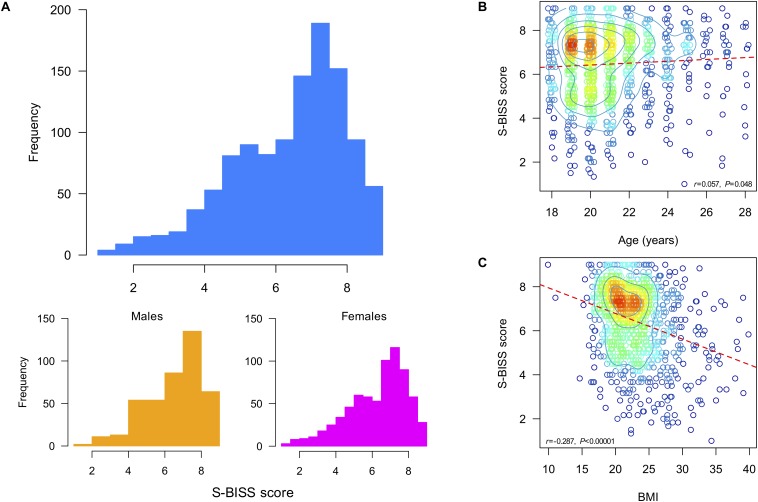
**(A)** Histogram of the S-BISS score for the full sample (top) and by gender (bottom), and as a function of **(B)** age, in years, and **(C)** BMI. S-BISS = Spanish Body Image State Scale; BMI = Body Mass Index.

Figure 2A shows the distribution of the average S-BISS score in our sample. The average S-BISS score was 6.46 ± 1.6 (range 1–9) and differed between males and females (6.62 ± 1.54 vs. 6.36 ± 1.63, *W* = 163260, *p* = 0.016; effect size [$\hat{\phi }$] = 2.68; Table 1). Using the Pearson’s correlation coefficient, we found that higher S-BISS scores are statistically associated with higher age (Figure 2B) and lower S-BISS scores with higher BMI (Figure 2C).

**TABLE 1:** Mean (*M*) and standard deviation (*SD*) of the S-BISS score by gender and BMI.

	**Gender**				**BMI category**							
					**Underweight**		**Normal weight**		**Overweight**		**Obese**	
	**Male**		**Female**		**(BMI < 18.5)**		**(18.5 ≤ BMI < 25)**		**(25 ≤ BMI < 30)**		**(BMI ≥ 30)**	
	
***N***	419		718		115		769		197		56	
	***M***	***SD***	***M***	***SD***	***M***	***SD***	***M***	***SD***	***M***	***SD***	***M***	***SD***
	
S-BISS score	6.62	1.54	6.36	1.63	7.03	1.35	6.63	1.46	5.83	1.79	5.12	1.87

*A Kruskal–Wallis tests showed that the S-BISS score differed by BMI categories ($\chi_{3}^{2}$ = 76.65, *p* < 0.0001). Abbreviations as in Figure 2.*

**TABLE 2:** Results of the confirmatory factor analysis applied to the BISS scale in a Colombian population.

**Model**	**Structure**	χ^2^ **(df)**	**AIC**	**BIC**	**TLI**	**CFI**	**RMSEA**
1	One factor model	231.81 (9)	23029.9	23090.3	0.907	0.956	0.148
2	One factor model with Q5∼Q6	124.83 (8)	22924.9	22990.4	0.950	0.977	0.113
3	One factor model with Q4∼Q6 and Q5∼Q6	71.79 (7)	22873.9	22944.4	0.972	0.987	0.090
4	One factor model with Q4∼Q6, Q4∼Q5, and Q5∼Q6	10.01 (6)	**22814.1**	**22889.6**	**0.998**	**0.999**	**0.024**

**χ*^2^, Test statistic; df = degrees of freedom; AIC, Akaike’s information criterion; BIC, Bayesian information criterion; TLI, Tucker Lewis index; CFI, comparative fit index; RMSEA, root mean square error of approximation. In models 2, 3, and 4 the “∼” sign means “correlated.” Thus, “Q5∼Q6” implies that items 5 and 6 in the BISS scale are allowed to be correlated in the model. Best performance indicators are in bold. In all models, *p* < 0.00001 for the *χ*^2^statistic.*

**TABLE 3:** Summary statistics, reliability and factor (pattern) matrix for items of the S-BISS score.

**Item**		***M* (*SD*)**	**Eigenvalue^*a*^**	**α (*SD*)**	**α^–^**	**r**	**r^–^**	**Factor loading**	***h*2**	***u*2**
	**Right now I feel…**									
Q1	…with my physical appearance	6.6 (1.9)	4.37	0.89 (0.89)	0.90	0.88	0.84	0.882	0.778	0.222
Q2	…with my body size and shape	6.5 (1.9)	0.54	0.90 (0.89)	0.90	0.88	0.85	0.887	0.786	0.214
Q3	… with my weight	6.1 (2.2)	0.36	0.85 (0.84)	0.91	0.80	0.77	0.806	0.650	0.350
Q4	… physically attractive	6.7 (1.7)	0.31	0.84 (0.85)	0.91	0.80	0.77	0.808	0.652	0.348
Q5	… about my looks than I usually feel	6.6 (1.8)	0.26	0.80 (0.81)	0.91	0.75	0.72	0.750	0.562	0.438
Q6	that I look … than the average person looks	6.3 (1.7)	0.16	0.83 (0.84)	0.91	0.79	0.75	0.788	0.621	0.379

*^*a*^Shown in Figure 2B. *M*, mean; *SD*, standard deviation; α, Cronbach’s α; α^–^, Cronbach’s α when the corresponding item is dropped; *r*, Correlation with the total S-BISS score; *r*^–^, Correlation with the total S-BISS score when the item when the corresponding item is dropped; *h*^2^, proportion of variance of each item that can be explained by Factor 1; *u*, 1-*h*^2^. S-BISS, Spanish Body Image State Scale.*

Furthermore, there is a statistically significant gender (*F*_1_,_1133_ = 5.81, *p* = 0.0161) and a BMI (*F*_1_,_1133_ = 124.39, *p* < 0.0001) effect on the S-BISS score after correction for age is performed. Table 1 provides norm values of the mean S-BISS scores by gender and BMI categories, following the World Health Organization [WHO] (2000) guidelines. A Kruskal–Wallis tests revealed that the average S-BISS score statistically differs across BMI categories ($\chi_{3}^{2}$ = 75.65, *p* < 0.0001).

### Convergent and Divergent Validity

The S-BISS correlated negatively with the BSQ-8c (*r* = −0.577, *p* < 0.001; Supplementary Figure 1), indicating that a low state body image (or low state body satisfaction) as assessed with the S-BISS is related to higher BD on a cognitive-affective level. In addition, a negative correlation between the S-BISS and the BSI-53 was found (*r* = −0.331, *p* < 0.001; Supplementary Figure 1), indicating that lower S-BISS scores are generally associated with more psychopathology. Furthermore, the S-BISS score of participants without self-reported eating disorders (*n* = 1122) was statistically significantly lower in individuals who were currently on a diet (*n* = 228) compared to those who did not report (*n* = 894) dieting behavior (6.09 ± 1.71 vs. 6.56 ± 1.56; *t*_1135_ = −3.98, $\hat{\phi }=3.77$, *p* < 0.001).

## Discussion

In this study, we assessed the psychometric properties and validity of the Spanish translation of a state measure of body image (S-BISS) in a Colombian population. As assumed by the authors of the original BISS version (Cash et al., 2002), a one-factor structure was derived in the translated questionnaire. Regarding the factorial structure of the S-BISS, both exploratory and confirmatory factor analyses identified a single factor solution. Prior to conducting our exploratory factor analysis, we proposed four suitable models when performing confirmatory factor analysis. These models allowed several degrees of correlation between items of the S-BISS to better explain the factor structure. Interestingly, a model with three correlated items fitted the data best, suggesting that the S-BISS questionnaire is not an orthogonal entity. The latter result is a significant contribution of this study, as to the best of our knowledge, the factor structure of the BISS has not been examined either in English or in a translated version.

Our reliability analysis reported a sufficient scale score reliability for the full sample and when separated by gender. Even when specific items were dropped from the scale, the scale score reliability coefficient remains within acceptable limits, which suggests that the S-BISS scale can be considered a suitable psychometric instrument to assess body image in a Colombian population. Interestingly, our reliability indices were considerably higher compared to the English BISS version which reported Cronbach’s α between 0.62 and 0.77 in the neutral condition (Cash et al., 2002).

The S-BISS showed good convergent validity with other body image measures (in consideration of the body satisfaction score of the S-BISS, lower S-BISS scores were associated with higher measures of body dissatisfaction) (Supplementary Figure 1). This indicates that momentary body dissatisfaction measured on the S-BISS is associated with trait body dissatisfaction measured with the BSQ-8. Although this negative correlation appears to be counter-intuitive, it is due to different scale ranges of the S-BISS (Cash et al., 2002) and in the BSQ-8 high values indicate body dissatisfaction (Cooper et al., 1987). Convergent validity with a trait body image measurement and a general psychopathology questionnaire were as expected and statistically significant differences between BMI categories and dieters/non-dieters were found. A positive association between age and the S-BISS score was obtained, although the correlation coefficient was extremely small and thus without practical relevance. In addition, a negative and meaningful association between BMI and the S-BISS was observed indicating that individuals with a higher BMI experience themselves as momentarily less satisfied with their body image, which is in line with general findings regarding the relation between BMI and body image, especially in obesity (Markey and Markey, 2005; Weinberger et al., 2016). However, this correlation was lower than in the original BISS publication (Cash et al., 2002), which might be due to sample and/or cultural differences. According Holmqvist and Frisén (2010), body satisfaction is influenced by sociocultural factors like parameters about attractiveness and body shape. Other factors involve are media and peers’ pressures on appearance ideal internalization. All of them are related with own physical assessment (Llorente et al., 2013; Cortez et al., 2016). Furthermore, the BMI values in our sample were self-reported and there is evidence that self-reported height and weight might be biased especially for individuals with a higher body weight. In line with the original publication but also to a lower extent, higher S-BISS scores in our sample were also associated with lower trait body dissatisfaction on the cognitive-affective dimension, as measured with the BSQ-8c. The differences between the original instrument and our translation might be partially explained by sample differences, as the original sample was only comprised of United States American psychology students, whereas in our study a more diverse sample was obtained regarding age and study field.

Several limitations were noted. For instance, we did not assess the test-retest reliability of the instrument in our sample, nor did we assess state body image differences in experimental situations or daily life. Thus, it can be presumed that the S-BISS will reveal substantial situational changes of body image. Our study can merely be perceived as a starting point in the evaluation of the S-BISS, providing insight in its basic psychometrics as, for example, factor structure and estimated internal consistency. Studies to detect state body image in a satisfactory manner, for example in ecological momentary assessment (EMA) or experimental studies, are clearly needed. A second intrinsic limitation about the validation of the S-BISS is related to the number of items comprising the original questionnaire. As the S-BISS instrument only contains six items, both the exploratory and confirmatory factor analyses are limited as a minimum of three items are needed to reliably identify any factor. Future factor analyses of the S-BISS in other Colombian and/or Latin American samples would definitely help to better understand the factor structure derived herein, and potential modifications in other regions.

Furthermore, our analyses relied on self-reported BMI and dieting behaviors. Future studies should investigate if the S-BISS detects differences in body satisfaction between a clinical sample with eating disorders and a healthy control sample. It can be assumed that differences between a homogenous clinical sample and healthy participants would be greater than any differences reported in this study, and differences between BMI groups might be more pronounced. Thus, these findings of our study have to be considered with caution.

A third limitation of our study is that participants were not exposed, during a follow up experiment, to any other situational contexts. However, considering the high response rate of our current study and the good properties of the questionnaire, to use of our S-BISS version for experiments with context factors or EMA studies by other research groups in Spanish-speaking Latin America countries is yet to be explored.

A significant strength of our study is its large and diverse sample. We were able to obtain a large sample size of young Colombian individuals with diverse educational backgrounds. Although almost 80% of our participants were currently enrolled as university students, only 40% were psychology students. This strengthens the generalizability of our results compared to other studies based on psychology undergraduate students only. Additionally, the excellent reliability measures and the clear factorial structure of the S-BISS, which could in principle be replicated compared to the original version, increase the usability of the questionnaire.

Our finding that the S-BISS correlated strongly with trait measures of body image indicate that the S-BISS, in addition to being designed as a state measurement, also captures at least partially a trait component of body satisfaction. Assuming that the S-BISS truly assesses momentary body satisfaction, a high degree of within-person variance from one measurement to another in diverse situational settings could be expected. For example, an experimental study could examine momentary body image with the S-BISS prior and posterior to and after several situations with theoretical high influence on body image, such as viewing oneself in a mirror, a high-calorie meal, or imagining walking in bathing clothes at a beach [e.g., as in Cash et al. (2002)]. Another approach to further examine the state validity and reliability of the S-BISS could be applying the questionnaire on several occasions in a naturalistic setting during the daily life of participants in an EMA approach (Shiffman et al., 2008). Previous EMA studies using the original version of the BISS showed good validity of the measure in a daily life study (Fuller-Tyszkiewicz et al., 2015, 2018). Hence, it would be interesting to assess whether a Latin American population differs from Western samples regarding the within-person variability over a day or across situations with a high influence on body image, given that these populations differ in their trait body image and body satisfaction to at least some extent.

## Conclusion

Overall, our results suggest that the S-BISS is a suitable, valid and reliable instrument to assess body satisfaction in Colombian populations. By extension, S-BISS might be used in other Spanish-speaking countries as well. However, the scores themselves should be carefully checked and properly adapted for the cultural and linguistic characteristics specific to these populations. Future studies should include the use of the S-BISS in other Spanish-speaking countries and address the potential effect of cultural differences on the S-BISS score. With the S-BISS, there is now a validated instrument to assess cultural moderators on state body image in varying contexts. For example, it could be interesting to assess whether state body satisfaction at the beach is higher for Colombians from the coastal regions compared to Colombians from the capital city, or if ethnicity (e.g., Afro-Colombian, predominantly Hispanic, Indigenous) is a moderator on state body image in different contexts. However, the validity and reliability of the questionnaire, when applied in diverse situational contexts, remains to be explored.

## Data Availability Statement

The datasets generated for this study are available on request from the corresponding authors.

## Ethics Statement

The studies involving human participants were reviewed and approved by the Universidad del Norte, Barranquilla, Colombia (No. 155/2017) and from the ethical review board of the local medical association in Mainz, Germany (No. 837.336.16/10655). The patients/participants provided their written informed consent to participate in this study.

## Author Contributions

DK and FH: conceptualization. DK: data curation and supervision. JV and DK: formal analysis. MM, DM-R, FH, HP, and DK: investigation. JV, FH, and DK: methodology. MM, DM-R, and DK: project administration. MM and DM-R: resources. MM, LT, JV, and DK: writing – original draft. MM, LT, DM-R, JV, MM-B, FH, HP, and DK: writing – reviewing and editing.

## Conflict of Interest

The authors declare that the research was conducted in the absence of any commercial or financial relationships that could be construed as a potential conflict of interest.

## Footnotes

1 https://www.project-redcap.org/
