# Supplementary material for: The Spanish Body Image State Scale: Factor Structure, Reliability and Validity in a Colombian Population
Source: Front Psychol. 2019 Nov 22;10:2553. doi: 10.3389/fpsyg.2019.02553 (PMC6883919; doi:10.3389/fpsyg.2019.02553)

Spanish Body Image States Scale – Escala del estado de la imagen corporal (versión española)

Descripción: con el objetivo de abordar la necesidad de una medida psicométricamente sólida del estado evaluativo/afectivo de la imagen corporal de las personas, el autor desarrolló y validó la Spanish Body Image States Scale (S-BISS, Escala del Estado de la Imagen Corporal) que consta de seis ítems. El S-BISS tiene una consistencia interna aceptable. Como evidencia de su validez convergente, el S-BISS se correlaciona apropiadamente con varias mediciones de rasgos de la imagen corporal. Es sensible a reacciones en contextos situacionales positivos versus negativos. Las diferencias entre los sexos reflejan las esperadas según la literatura. La validez de constructo ha sido confirmada a través de un experimento sobre la reactividad diferencial a la información relacionada con la apariencia en función del nivel de disfuncionalidad de la imagen corporal. El S-BISS es una medida única y muy necesaria, con utilidad potencial tanto en la investigación como en el trabajo clínico.

Puntuación del S-BISS: El resultado es la media compuesta de los seis ítems de 9 puntos. El cuestionario debe ser puntuado de modo que los puntajes bajos reflejen estados más negativos de la imagen corporal y los puntajes altos reflejen estados más positivos. Antes de tomar la media de los seis ítems, invierta los ítems 2, 4 y 6. La puntuación inversa requiere recodificar los valores en estos tres ítems de la siguiente manera: 1 = 9, 2 = 8, 3 = 7, 4 = 6, 6 = 4, 7 = 3, 8 = 2, 9 = 1.

Para detalles estadísticos de la versión inglés del BISS, ver Cash, T.F., Fleming, E.C., Alindogan, J., Steadman, L., & Whitehead, A. (2002). Beyond body image as a trait: The development and validation of the Body Image States Scale. Eating Disorders: The Journal of Treatment & Prevention, 10, 103-113.

Al administrar el S-BISS, asegúrese de no imprimir esta página.

Cuestionario S-BISS

Para cada uno de los elementos siguientes marque la casilla junto a la declaración que mejor describe cómo se siente AHORA MISO, EN ESTE MOMENTO. Lea los ítems con cuidado para asegurarse de que la declaración que elija describe con exactitud y honestidad cómo se siente ahora mismo.

1. En este momento me siento ...

   Extremadamente insatisfecho con mi apariencia física

   Mayormente insatisfecho con mi apariencia física

   Moderadamente insatisfecho con mi apariencia física

   Ligeramente insatisfecho con mi apariencia física

   Ni satisfecho ni insatisfecho con mi apariencia física

   Ligeramente satisfecho con mi apariencia física

   Moderadamente satisfecho con mi apariencia física

   Mayormente satisfecho con mi apariencia física

   Extremadamente satisfecho con mi apariencia física

2. En este momento me siento ...

   Extremadamente satisfecho con el tamaño y la forma de mi cuerpo

   Mayormente satisfecho con el tamaño y la forma de mi cuerpo

   Moderadamente satisfecho con el tamaño y la forma de mi cuerpo

   Ligeramente satisfecho con el tamaño y la forma de mi cuerpo

   Ni satisfecho ni insatisfecho con el tamaño y la forma de mi cuerpo

   Ligeramente insatisfecho con el tamaño y la forma de mi cuerpo

   Moderadamente insatisfecho con el tamaño y la forma de mi cuerpo

Mayormente insatisfecho con el tamaño y la forma de mi cuerpo

Extremadamente insatisfecho con el tamaño y la forma de mi cuerpo

3. En este momento me siento ...

Extremadamente insatisfecho con mi peso

Mayormente insatisfecho con mi peso

Moderadamente insatisfecho con mi peso

Ligeramente insatisfecho con mi peso

Ni satisfecho ni insatisfecho con mi peso

Ligeramente satisfecho con mi peso

Moderadamente satisfecho con mi peso

Mayormente satisfecho con mi peso

Extremadamente satisfecho con mi peso

4. En este momento me siento ...

Extremadamente atractivo físicamente

Muy atractivo físicamente

Moderadamente atractivo físicamente

Ligeramente atractivo físicamente

Ni atractivo ni poco atractivo

Ligeramente poco atractivo físicamente

Moderadamente poco atractivo

Muy poco atractivo físicamente

Extremadamente poco atractivo físicamente

5. Ahora mismo me siento ...

Muchísimo peor acerca de mi apariencia de lo que normalmente me siento

Mucho peor acerca de mi apariencia de lo que normalmente me siento

Algo peor acerca de mi apariencia de lo que normalmente me siento

Sólo un poco peor acerca de mi apariencia de lo que normalmente me siento

Relativamente igual acerca de mi apariencia de lo que normalmente me siento

Sólo un poco mejor acerca de mi apariencia de lo que normalmente

me siento

Algo mejor acerca de mi apariencia de lo que normalmente me siento

Mucho mejor acerca de mi apariencia de lo que normalmente me siento

Muchísimo mejor acerca de mi apariencia de lo que normalmente me siento

6. En este momento siento que me veo ...

Muchísimo mejor que la persona promedio

Mucho mejor que la persona promedio

Algo mejor que la persona promedio

Sólo un poco mejor que la persona promedio se ve

Aproximadamente igual que la persona promedio

Sólo un poco peor que la persona promedio se ve

Un poco peor que la persona promedio

Mucho peor que la persona promedio

Muchísimo peor que la persona promedio

Supplementary Figure 1. Scatterplot for the S-BISS score as a function of the **(a)** BSQ and **(b)** BSI scores. Our results indicate that lower values of the S-BISS are associated with more trait body dissatisfaction on a cognitive-affective level and more psychopathology, respectively. BSQ = Body Shape Questionnaire; BSI = Brief Symptom Inventory.

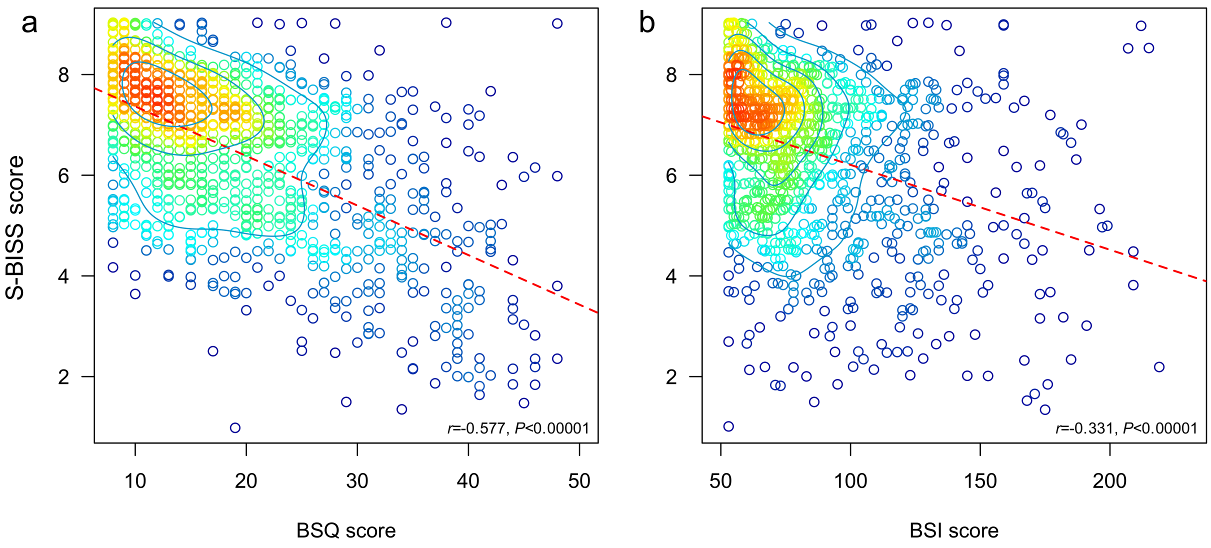

Supplement: Supplementary file 1 [file Data_Sheet_1.PDF]
